# Effects of astigmatic defocus on binocular contrast sensitivity

**DOI:** 10.1371/journal.pone.0202340

**Published:** 2018-08-14

**Authors:** Yumi Hasegawa, Takahiro Hiraoka, Shinichiro Nakano, Fumiki Okamoto, Tetsuro Oshika

**Affiliations:** 1 Department of Ophthalmology, Faculty of Medicine, University of Tsukuba, Ibaraki, Japan; 2 Department of Ophthalmology, Ryugasaki Saiseikai Hospital, Ibaraki, Japan; National Yang-Ming University Hospital, TAIWAN

## Abstract

**Purpose:**

To determine the effects of astigmatism on contrast sensitivity (CS).

**Methods:**

Eighteen normal volunteers (30.5 ± 6.0 [mean ± SD] years) were recruited. After correcting each refractive error by spectacles, against-the-rule (ATR) or with-the-rule (WTR) astigmatism of +1.00, +2.00 and +3.00 D was intentionally produced in both eyes, and then binocular CS was measured. The cylindrical addition of different powers (+1.00–+3.00 D) was compensated with spherical lenses so that the spherical equivalent refraction became zero in each eye. Subsequently, the above cylindrical addition was monocularly induced, and binocular CS was measured again. The relation between CS and astigmatic power, axis, and monocular or binocular astigmatism was investigated.

**Results:**

With binocular ATR and WTR astigmatism, increases in astigmatic power significantly correlated with decreases in the area under the log contrast sensitivity function (AULCSF). With monocular astigmatic defocus, astigmatic power addition did not affect AULCSF. With binocular astigmatic defocus of high-power (+2.00 and +3.00 D), ATR astigmatism deteriorated AULCSF more than WTR astigmatism. In a comparison between binocular and monocular astigmatic defocus, CS was significantly worse with binocular astigmatic defocus than with monocular astigmatic defocus at higher spatial frequencies regardless of astigmatic power.

**Conclusions:**

Binocular astigmatic defocus deteriorates CS depending on the amount of astigmatic power. ATR astigmatism reduces CS more than WTR astigmatism dose. In addition, binocular astigmatic defocus affects CS more severely than monocular astigmatic defocus especially at high spatial frequencies.

## Introduction

It has been known that uncorrected monocular astigmatism deteriorates monocular visual acuity, contrast sensitivity (CS), reading performance and functional visual acuity [1–6]. Little is known, however, about the effect of uncorrected astigmatism on binocular visual function in various conditions such as binocular, monocular, against-the-rule (ATR) or with-the-rule (WTR) astigmatism. In particular, only limited studies have reported on the effect of monocular uncorrected astigmatism on binocular visual function [2,7,8]. To our knowledge, no research has been conducted regarding the effect of monocular uncorrected astigmatism on CS.

Aniso-astigmatism was uncommon in the natural history [9–11]. Linke et al. reported that 2.59% patients have 1.75 diopters (D) and more aniso-astigmatism in myopic refractive surgery candidates [10]. In recent years, toric intraocular lens (IOL), which can eliminate or reduce postoperative astigmatism, has been widely applied in clinical practice. Astigmatism, however, is not always and completely removed in each eye. Some patients have residual binocular or monocular astigmatism after operation. Therefore, it is crucial to clarify the influences of monocular or binocular uncorrected astigmatism on binocular CS.

The purpose of this study was to investigate the effects of monocular and binocular astigmatism on binocular CS and also explore the influences of ATR and WTR astigmatism on binocular CS.

## Materials and methods

### Ethics statement

This study was conducted in accordance with the tenets of the Declaration of Helsinki, and the study protocol was approved by the Ethics Committee University of Tsukuba Hospital. Study participants provided written informed consent.

### Subjects

Healthy adult volunteers who had no ophthalmic disease other than refractive errors were enrolled in this study. The inclusion criteria included best-corrected visual acuity of 0.00 logMAR or better, Titmus Stereo Test of 40 seconds of arc or better, spherical equivalent refraction up to 6.00 diopters (D) and refractive astigmatism up to 0.75 D.

### Examination

First, after correcting each refractive error by spectacles binocular distance CS was measured as the baseline. Subsequently binocular CS was measured with induced different astigmatisms. ATR or WTR astigmatism of +1.00, +2.00 or +3.00 D was intentionally produced in both eyes, and then, the above cylindrical addition was monocularly induced (i.e.; no astigmatism in fellow eye). The cylindrical addition of different powers (+1.00–+3.00 D) was compensated with spherical lenses so that the spherical equivalent refraction became zero for each eye. The order of cylindrical induction with different powers and axes was randomly determined. CS was assessed at five spatial frequencies (1.5, 3, 6, 12 and 18 cycles per degree [cpd]) with the OPTEC 6500 Vision Tester^®^ (Stereo Optical Co., Inc, Chicago, IL). From the data obtained with the OPTEC 6500 Vision Tester^®^, the area under the log contrast sensitivity function (AULCSF) was calculated according to the methods of Applegate and associates [12]. We investigated AULCSF in relation to astigmatic power, axis and monocular or binocular astigmatism. In addition, the comparison log CS between monocular and binocular astigmatism was assessed in each spatial frequency.

### Statistical analyses

The mean and standard deviations were calculated for CS and other parameters. The Spearman correlation test was performed to determine the relationship between astigmatic powers and AULCSF. The Wilcoxon signed-ranks test was used to compare AULCSF between ATR and WTR astigmatism. Fisher's protected least-significant difference (PLSD) was performed to compare AULCSF among three astigmatic conditions (full correction for both eyes, monocular, and binocular astigmatism). The Wilcoxon signed-ranks test was also used to compare log CS between binocular and monocular astigmatic defocus in each spatial frequency. All tests were considered statistically significant if P < 0.05. The analyses were carried out with Stat View (version 5.0, SAS Inc., Cary, NC).

## Results

Eighteen subjects (11 men and 7 women) were enrolled in this study. The mean age was 30.5 ± 6.0 [mean ± SD] years (range 22 to 42 years). The mean spherical equivalent refraction was -1.53 ± 1.76 D (range 0.00 to -6.00 D) and the mean refractive astigmatism was 0.17 ± 0.26 D (range 0.00 to 0.75 D).

**Fig 1:**
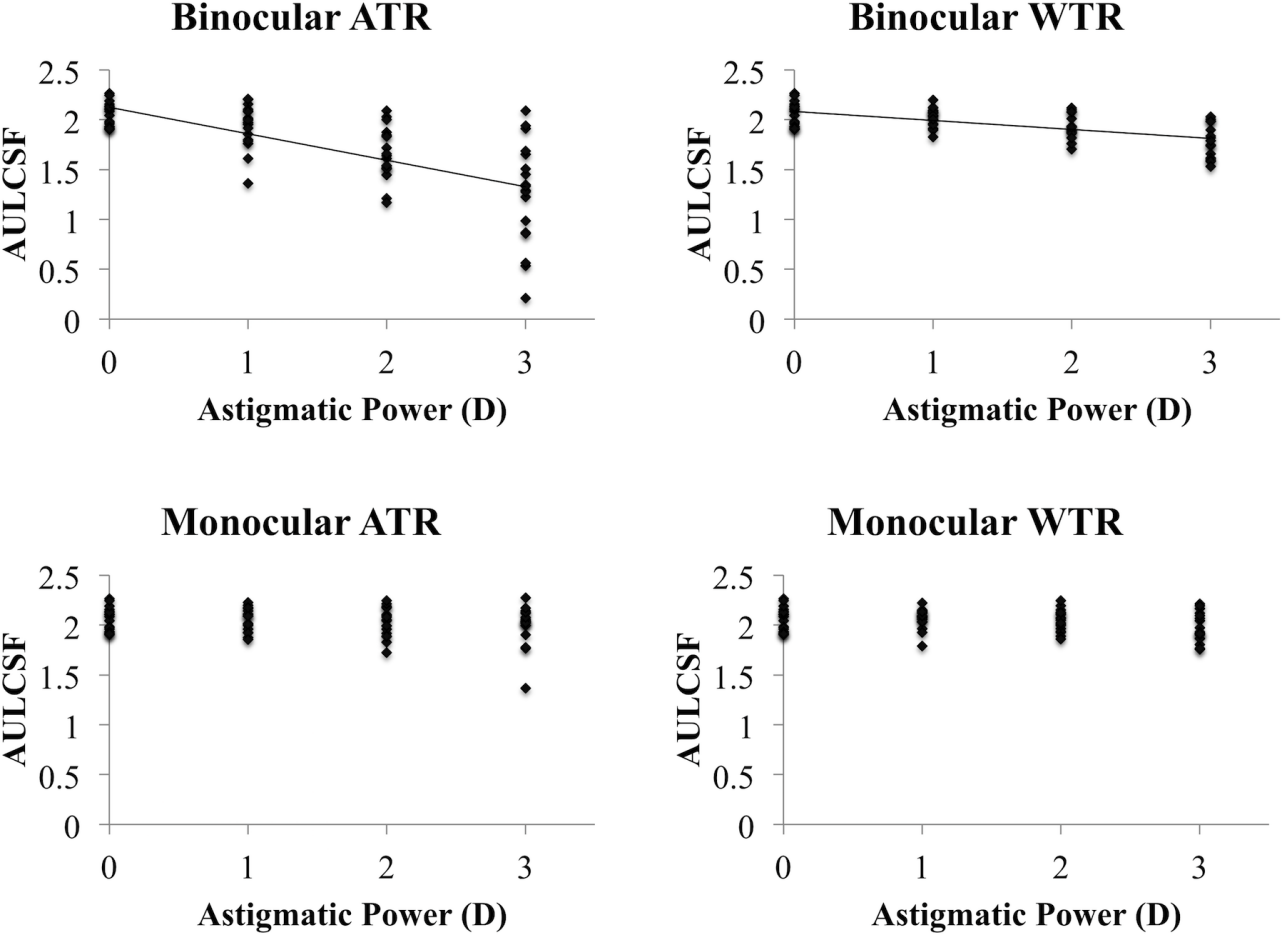
Relationship between astigmatic powers and contrast sensitivity (CS). In binocular astigmatism, area under the log contrast sensitivity function (AULCSF) decreased as astigmatic power increased (P < 0.0001, Spearman correlation test). In contrast, astigmatic power did not affect AULCSF in monocular astigmatism. (ATR = against-the-rule, WTR = with-the-rule).

**Fig 2:**
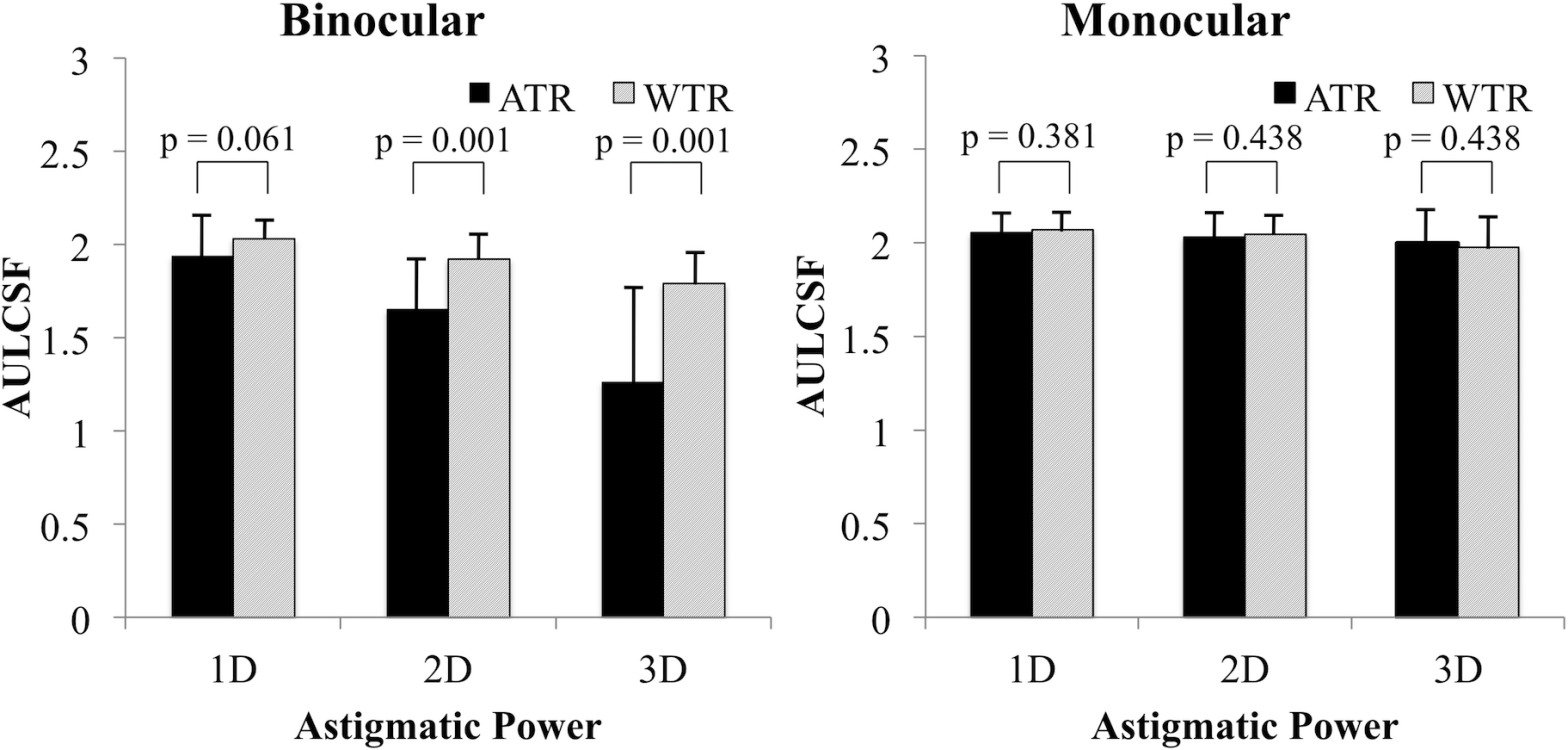
Comparison of area under the log contrast sensitivity function (AULCSF) between against-the-rule (ATR) and with-the-rule (WTR) astigmatism. In binocular +2.00 and +3.00 D astigmatic defocus, ATR astigmatism resulted in significantly worse AULCSF than WTR astigmatism. In monocular astigmatic defocus, AULCSF showed no significant differences between ATR and WTR astigmatism.

**Fig 3:**
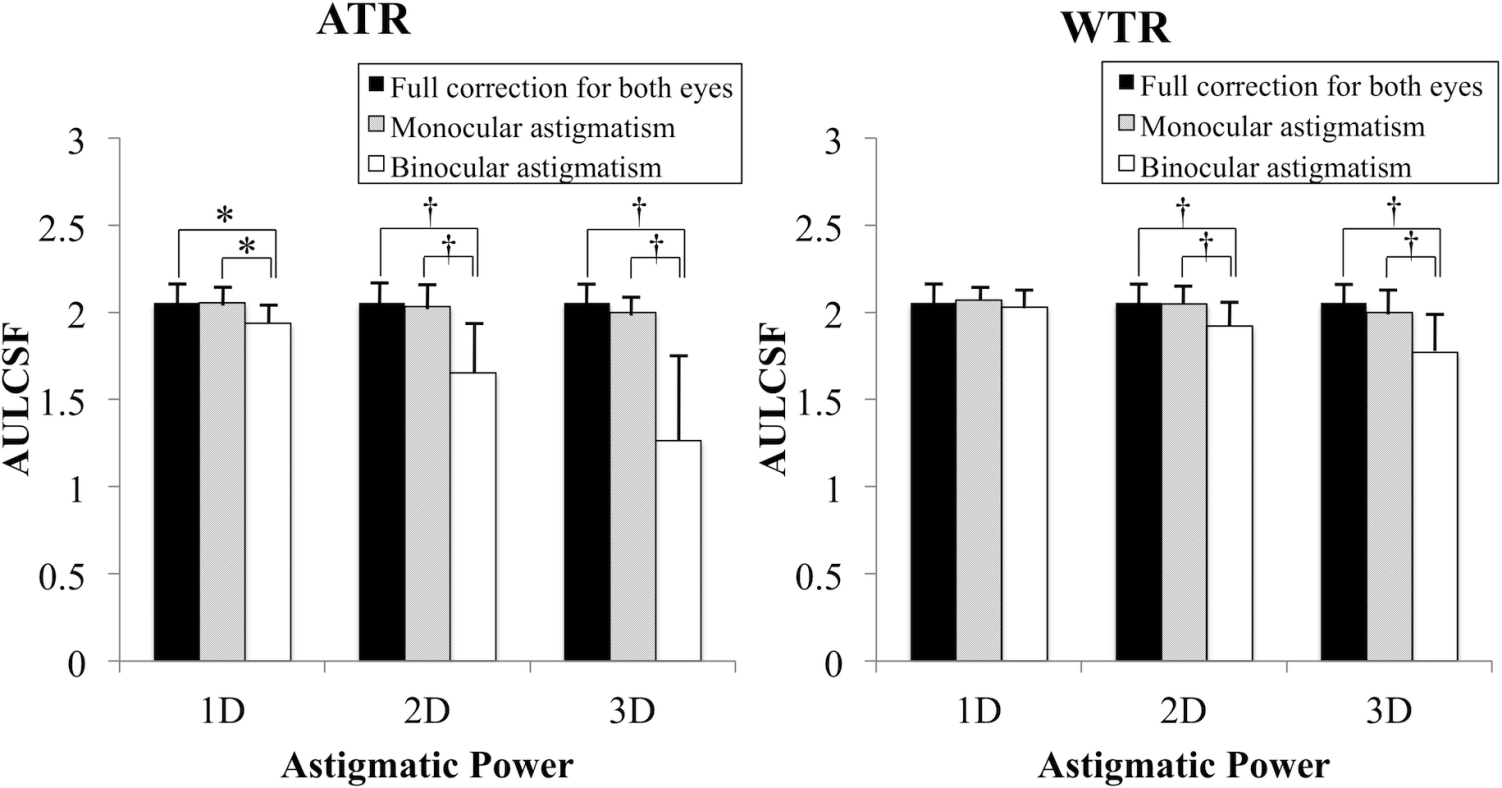
Comparison of area under the log contrast sensitivity function (AULCSF) among three astigmatic conditions. In against-the-rule (ATR) astigmatic defocus or +2.00 D and +3.00 D with-the-rule (WTR) astigmatic defocus, AULCSF under binocular astigmatic conditions was significantly worse than that under other astigmatic conditions. (*P < 0.05; †P < 0.01, Fisher's protected least-significant difference).

**Fig 4:**
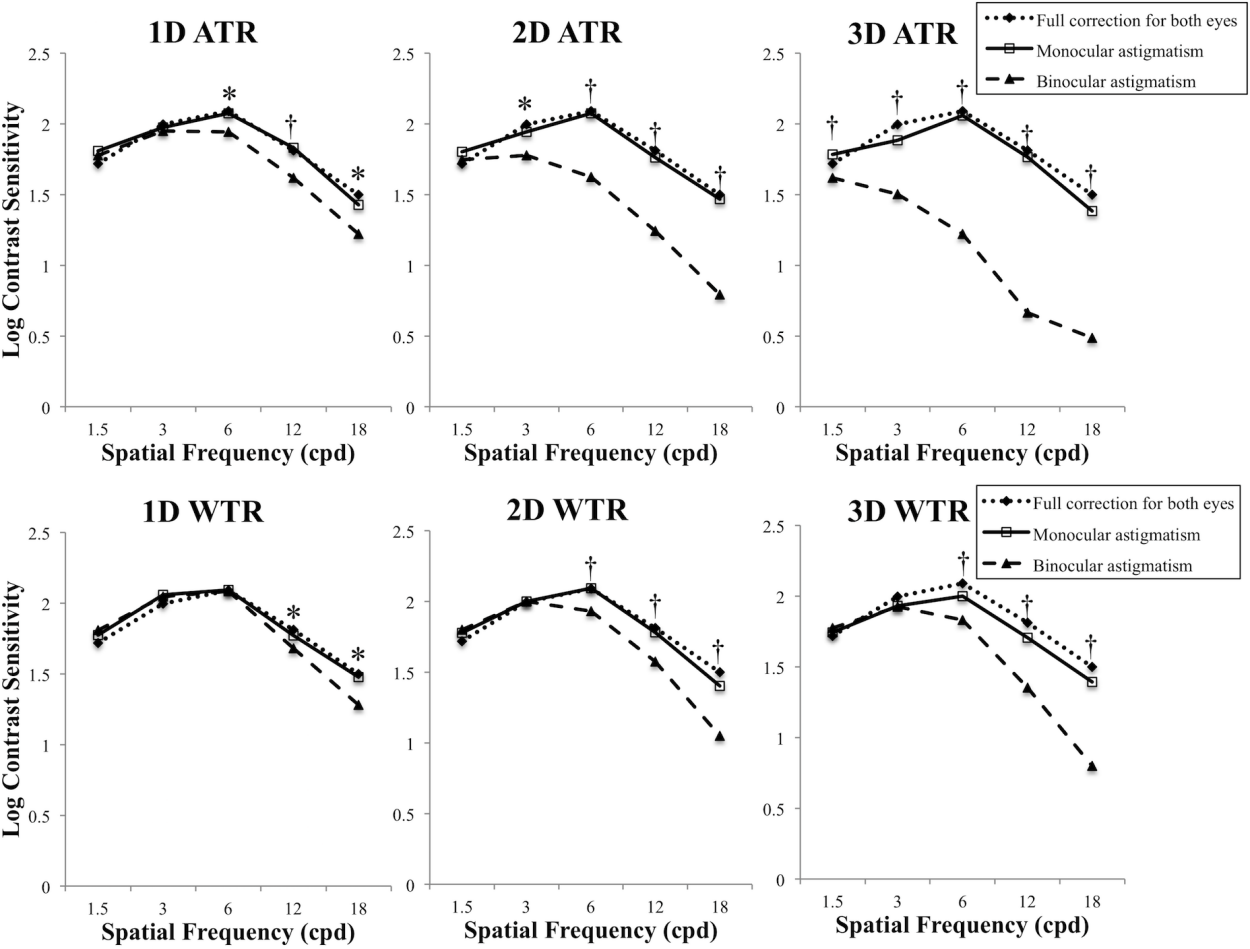
Comparison of log contrast sensitivity (CS) between binocular and monocular astigmatic defocus in each spatial frequency. When we compared log CS in each spatial frequency, CS under binocular conditions was significantly worse than that under monocular conditions at higher spatial frequencies (especially at 12 and 18 cpd) regardless of astigmatic power. (*P < 0.05; †P < 0.01, Wilcoxon signed-ranks test) (ATR = against-the-rule, WTR = with-the-rule).

Table 1 shows the mean log CS and AULCSF. Fig 1 shows the relationship between astigmatic powers and CS (AULCSF). In binocular ATR and WTR astigmatic conditions, AULCSF decreased as astigmatic power increased (r_s_ = -0.71, P < 0.0001 for ATR; r_s_ = -0.59, P < 0.0001 for WTR). In contrast, astigmatic power did not affect AULCSF in monocular astigmatic conditions. Fig 2 shows a comparison of AULCSF between ATR and WTR astigmatism in binocular or monocular astigmatic defocus. In binocular high-power (+2.00 and +3.00 D) astigmatic defocus, ATR astigmatism resulted in significantly worse AULCSF than WTR astigmatism (P = 0.001). In monocular astigmatic defocus, there were no significant differences in AULCSF between ATR and WTR astigmatism. Fig 3 indicates a comparison of AULCSF among three astigmatic conditions (full correction for both eyes, monocular, and binocular astigmatism). In ATR astigmatic defocus, AULCSF under binocular astigmatic conditions was significantly worse than that under monocular astigmatic conditions in all powers (P = 0.033 for +1.00 D, P < 0.0001 for +2.00 D and +3.00 D). A similar result was observed when we made a comparison between the conditions under binocular astigmatic defocus and full correction for both eyes. (P = 0.033 for +1.00 D, P < 0.0001 for +2.00 D and +3.00 D). In high power (+2.00 D and +3.00 D) WTR astigmatic defocus, AULCSF under binocular astigmatic conditions was significantly worse than that under monocular astigmatic conditions (P = 0.002 for +2.00 D, P = 0.003 for +3.00 D). A similar result was observed when we compared AULCSF between the conditions under binocular astigmatic defocus and full correction for both eyes. (P = 0.001 for +2.00 D, P < 0.0001 for +3.00 D). There were no significant differences in AULCSF between the conditions under monocular astigmatic defocus and full correction for both eyes, regardless of the astigmatic powers and axes. Fig 4 demonstrates a comparison of log CS between binocular and monocular astigmatic defocus in each spatial frequency. In +1.00 D ATR astigmatic defocus, CS under binocular astigmatic conditions was significantly worse than that under monocular astigmatic conditions at 6, 12 and 18 cpd (P = 0.016, P = 0.002, P = 0.014, respectively). In +2.00 D ATR astigmatic defocus, a similar result was observed at 3, 6, 12 and 18 cpd (P = 0.021, P = 0.003, P = 0.0002, P = 0.0002, respectively). Also in +3.00 D ATR astigmatic defocus, a similar result was observed at 1.5, 3, 6, 12 and 18 cpd (P = 0.003, P = 0.002, P = 0.0003, P = 0.0002, P = 0.003, respectively). In +1.00 D WTR astigmatic defocus, CS under binocular astigmatic conditions was significantly worse than that under monocular astigmatic conditions at 12 and 18 cpd (P = 0.029 and P = 0.012, respectively). In +2.00 D WTR astigmatic defocus, a similar result was observed at 6, 12 and 18 cpd (P = 0.003, P = 0.002, P = 0.0006, respectively). Also in +3.00 D WTR astigmatic defocus, a similar result was observed at 6, 12 and 18 cpd (P = 0.01, P = 0.0007, P = 0.0003, respectively).

**Table 1:** The log CS and AULCSF when induced each astigmatic type; astigmatic power, axis and monocular or binocular.

Astigmatism induction	Log CS					AULCSF
	1.5 cpd	3 cpd	6 cpd	12 cpd	18 cpd	
Full correction	1.72 ± 0.17	2.00 ± 0.13	2.09 ± 0.11	1.81 ± 0.20	1.50 ± 0.19	2.05 ± 0.12
Binocular ATR 1D	1.78 ± 0.16	1.95 ± 0.18	1.94 ± 0.29	1.62 ± 0.26	1.22 ± 0.43	1.94 ± 0.22
2D	1.75 ± 0.25	1.78 ± 0.27	1.62 ± 0.33	1.24 ± 0.37	0.79 ± 0.45	1.65 ± 0.26
3D	1.62 ± 0.21	1.50 ± 0.56	1.22 ± 0.64	0.67 ± 0.71	0.49 ± 0.57	1.26 ± 0.52
Monocular ATR 1D	1.81 ± 0.14	1.97 ± 0.14	2.08 ± 0.12	1.83 ± 0.15	1.43 ± 0.19	2.05 ± 0.11
2D	1.80 ± 0.14	1.94 ± 0.15	2.07± 0.16	1.76 ± 0.21	1.47 ± 0.17	2.05 ± 0.11
3D	1.78 ± 0.16	1.88 ± 0.48	2.06 ± 0.15	1.76 ± 0.18	1.38 ± 0.26	2.00 ± 0.20
Binocular WTR 1D	1.81 ± 0.13	2.05 ± 0.10	2.08 ± 0.09	1.68 ± 0.17	1.28 ± 0.24	2.03 ± 0.09
2D	1.80 ± 0.14	2.00 ± 0.08	1.93 ± 0.17	1.57 ± 0.20	1.05 ± 0.22	1.92 ± 0.12
3D	1.78 ± 0.16	1.92 ± 0.18	1.83 ± 0.17	1.35 ± 0.24	0.80 ± 0.39	1.79 ± 0.17
Monocular WTR 1D	1.78 ± 0.13	2.06 ± 0.05	2.09 ± 0.17	1.78 ± 0.15	1.48 ± 0.16	2.07 ± 0.10
2D	1.78 ± 0.17	2.00 ± 0.08	2.09 ± 0.17	1.78 ± 0.17	1.40 ± 0.16	2.05 ± 0.11
3D	1.74 ± 0.14	1.93 ± 0.12	2.00 ± 0.15	1.71 ± 0.21	1.39 ± 0.25	1.98 ± 0.14

Values are presented as mean ± standard deviation. CS = contrast sensitivity, AULCSF = area under the log contrast sensitivity function, cpd = cycles per degree, D = diopter. ATR = against-the-rule, WTR = with-the-rule

## Discussion

Several studies have proved that monocular astigmatic defocus reduces monocular CS [1,3]. Meanwhile, Wolffsohn et al. reported that binocular low-contrast distance visual acuity decreased with increasing uncorrected astigmatic power under the presence of binocular astigmatic defocus [13]. There has been no research, however, on the effect of uncorrected monocular astigmatism on binocular CS. In this study, under the presence of binocular astigmatic defocus, binocular CS decreased as astigmatic power increased. In contrast, under the presence of monocular astigmatic defocus, binocular CS did not decrease. In the present study, we for the first time revealed that monocular astigmatic defocus did not affect binocular CS. It means that monocular astigmatism is more tolerable in the refractive or cataract surgery.

In comparison with binocular WTR astigmatism, binocular ATR astigmatism resulted in significantly worse binocular CS. Wolffsohn et al. reported similar results that induced -3.00 D binocular oblique or ATR astigmatism resulted in worse binocular low-contrast distance visual acuity than WTR astigmatism [13]. In addition, Willis et al. showed that ATR astigmatism has significantly greater effects on reading performance than WTR astigmatism [14]. Based on these findings, ATR astigmatism affects visual function more negatively than WTR astigmatism under binocular astigmatic defocus. Some studies, however, presented the opposite results [15], or described that there was no statistically significant difference in visual function between ATR and WTR astigmatism [4–6,16]. Bradley et al. reported that the Vistech CS chart was rather insensitive to the effects of WTR than ATR astigmatic defocus, whereas the Pelli-Robson CS chart was very resistant to the effects of all types of astigmatic defocus [3]. In present study, we measured CS using the OPTEC 6500 Vision Tester^®^ with vertically striped charts which are also employed in the Vistech chart. In WTR astigmatism vertical lines are sharper for distance [16]. Therefore, it is possible that the characteristics of the CS chart affected these results. Table 2 shows the summary of visual function charts and the influence of the axis of astigmatism in each study.

**Table 2:** Summary of previous studies comparing the effect of axis on visual performance.

	Induction of astigmasitm	Visual performance	Target chart	Axis orientation
Wolffsohn^[^^13^^]^	Bionocular	Low contrast visual acuity	Letter	WTR > ATR = OBL
Willis^[^^14^^]^	Bionocular	Reading performance	Bailey-Lovie-word charts	WTR > ATR
Nanavaty^[^^15^^]^	Monocular	Far VA	Snellen	ATR > WTR = OBL
Kobashi^[^^4^^]^	Monocular	Far VA	Landolt-C	ATR = WTR > OBL
Remon^[^^5^^]^	Monocular	Far VA	Letters or Landolt-C	ATR = WTR = OBL
Watanabe^[^^6^^]^	Monocular	Low contrast visual acuity	CSV-1000LanC10%	ATR = WTR
Trindade^[^^16^^]^	Monocular	Far VA	Snellen	ATR = WTR
Bradley^[^^3^^]^	Monocular	CS	Vistech contrast	WTR > ATR
Bradley^[^^3^^]^	Monocular	CS	Pelli-Robson	ATR = WTR

VA = visual acuity, CS = contrast sensitivity, ATR = against-the-rule, WTR = with-the-rule, OBL = oblique

There has been no research on the effect of uncorrected monocular astigmatism on binocular CS. A comparison at each spatial frequency demonstrated that higher spatial frequencies were sensitive to astigmatic defocus regardless of astigmatic powers and axes. CS under binocular astigmatic conditions significantly decreased at higher spatial frequencies in both ATR and WTR astigmatic defocus, while no significant decrease was observed in binocular CS at lower spatial frequencies. Under monocular astigmatic defocus, no apparent decrease was observed in binocular CS in all spatial frequencies regardless of astigmatic powers and axes. Hence, if unilateral pseudophakic eye has residual astigmatism, astigmatism of the other eye should be aggressively corrected during cataract surgery and IOL implantation.

Our study had some limitations. First, we did not investigate the influence of adaptation to astigmatism. Adaptation to astigmatism is subjectively experienced already after 2 minutes, although it is unknown how long it lasts [17,18]. Second, we did not examine the influence of pupil size on CS. It is known that the effect of astigmatism on visual acuity is influenced by pupil size [19,20]. Further studies are needed to elucidate the relationship between pupil size and CS in various astigmatic conditions. Third, eye dominance is known to affect visual function [21], but we did not take this factor into consideration in this study.

## Conclusions

In conclusion, this is the first study that investigated the effect of binocular or monocular astigmatism on binocular CS in various conditions. Binocular astigmatic defocus deteriorated CS depending on the amount of astigmatic power. Moreover, the influence of ATR astigmatism on CS was larger than that of WTR astigmatism. Binocular astigmatic defocus affected binocular CS especially at high spatial frequencies, whereas monocular astigmatic defocus did not reduce binocular CS.

## Supporting information

S1 Table Subject’s information. (XLSX)

S2 Table Minimal dataset for astigmatism and contrast sensitivity. (ATR = against-the-rule, WTR = with-the-rule, AULCSF = area under the log contrast sensitivity function).
(XLSX)
